# Characterization of Fosfomycin and Nitrofurantoin Resistance Mechanisms in *Escherichia coli* Isolated in Clinical Urine Samples

**DOI:** 10.3390/antibiotics9090534

**Published:** 2020-08-24

**Authors:** Antonio Sorlozano-Puerto, Isaac Lopez-Machado, Maria Albertuz-Crespo, Luis Javier Martinez-Gonzalez, Jose Gutierrez-Fernandez

**Affiliations:** 1 Department of Microbiology, School of Medicine and PhD Program in Clinical Medicine and Public Health, University of Granada-ibs, 18016 Granada, Spain; asp@ugr.es (A.S.-P.); isloma@correo.ugr.es (I.L.-M.); albertuzmaria@correo.ugr.es (M.A.-C.); 2 Pfizer-University of Granada-Junta de Andalucía Centre for Genomics and Oncological Research (GENYO), 18016 Granada, Spain; luisjavier.martinez@genyo.es; 3 Laboratory of Microbiology, Virgen de las Nieves University Hospital-ibs, 18014 Granada, Spain

**Keywords:** *Escherichia coli*, fosfomycin, nitrofurantoin, antimicrobial resistance

## Abstract

Fosfomycin and nitrofurantoin are antibiotics of choice to orally treat non-complicated urinary tract infections (UTIs) of community origin because they remain active against bacteria resistant to other antibiotics. However, epidemiologic surveillance studies have detected a reduced susceptibility to these drugs. The objective of this study was to determine possible mechanisms of resistance to these antibiotics in clinical isolates of fosfomycin- and/or nitrofurantoin-resistant UTI-producing *Escherichia coli*. We amplified and sequenced *murA*, *glpT*, *uhpT*, *uhpA*, *ptsI*, *cyaA*, *nfsA*, *nfsB*, and *ribE* genes, and screened plasmid-borne fosfomycin-resistance genes *fosA3*, *fosA4*, *fosA5*, *fosA6*, and *fosC2* and nitrofurantoin-resistance genes *oqxA* and *oqxB* by polymerase chain reaction. Among 29 isolates studied, 22 were resistant to fosfomycin due to deletion of *uhpT* and/or *uhpA* genes, and 2 also possessed the *fosA3* gene. Some modifications detected in sequences of NfsA (His11Tyr, Ser33Arg, Gln67Leu, Cys80Arg, Gly126Arg, Gly154Glu, Arg203Cys), NfsB (Gln44His, Phe84Ser, Arg107Cys, Gly192Ser, Arg207His), and RibE (Pro55His), and the production of truncated NfsA (Gln67 and Gln147) and NfsB (Glu54), were associated with nitrofurantoin resistance in 15/29 isolates; however, the presence of *oqxAB* plasmid genes was not detected in any isolate. Resistance to fosfomycin was associated with the absence of transporter UhpT expression and/or the presence of antibiotic-modifying enzymes encoded by *fosA3* plasmid-mediated gene. Resistance to nitrofurantoin was associated with modifications of NfsA, NfsB, and RibE proteins. The emergence and spread of these resistance mechanisms, including transferable resistance, could compromise the future usefulness of fosfomycin and nitrofurantoin against UTIs. Furthermore, knowledge of the genetic mechanisms underlying resistance may lead to rapid DNA-based testing for resistance.

## 1. Introduction

The high incidence of urinary tract infections (UTIs) and their usually mild character means that most patients receive empirical antibiotic treatment. However, clinicians are now faced with major challenges due to multiple factors, including population aging, the presence of allergies or adverse reactions to antibiotics, an increased number of immunodepressed patients, and, especially, high rates of multi-resistant pathogens, which can cause therapeutic failure. A good alternative option may be to return to antibiotics such as fosfomycin and nitrofurantoin [1].

The characteristics of fosfomycin and nitrofurantoin make them especially useful for UTI treatment, including their rapid oral absorption, high urine concentration, and bactericide activity against a wide range of Gram-negative and Gram-positive bacteria. Both are first-line treatments for non-complicated UTIs of community origin [2]. They have also been reported to preserve their activity against multi-resistant microorganisms, especially uropathogenic enterobacteria such as *Escherichia coli* and extended-spectrum beta-lactamase-producing isolates [3], although these are usually less susceptible to fosfomycin and nitrofurantoin than are non-producers [4,5]. 

Currently, resistance to fosfomycin or nitrofurantoin is not common in our setting, and >85% of bacteria isolated in UTIs are susceptible to these antibiotics. Nonetheless, the gradual decrease in susceptibility to these drugs may lead to their contraindication as an empirical treatment in the future [1]. Any expansion of their clinical utilization would, therefore, require the adoption of epidemiological surveillance measures to detect the possible emergence of resistance [6]. With this background, the objective of this study was to explore possible molecular mechanisms underlying the resistance of clinical isolates of UTI-producing *E. coli* to fosfomycin and nitrofurantoin in our setting.

## 2. Methods 

### 2.1. Bacterial Isolates

The study included clinical isolates of fosfomycin- and/or nitrofurantoin-resistant *E. coli* with significant bacterial count selected from among urine cultures conducted for UTI analysis in the Microbiology Laboratory of Virgen de las Nieves University Hospital (Granada, Spain). They were identified by matrix-assisted laser desorption ionization-time of flight (MALDI-TOF) mass spectrometry (Bruker Daltonics, Bremen, Germany) as part of the routine microbiology laboratory workup [1]. A disk diffusion procedure (Kirby–Bauer) was also conducted on agar Mueller-Hinton plates, using McFarland 0.5 bacterial inoculum and disks with 200 μg fosfomycin supplemented with 50 μg glucose-6-phosphate (G6P) or disks with 300 μg nitrofurantoin. Each isolate was defined as "susceptible,” “intermediate,” or “resistant” according to the Clinical and Laboratory Standards Institute (CLSI) breakpoints [7]. For fosfomycin, an inhibition zone diameter ≥16 mm was considered susceptible, 13–15 mm intermediate, and ≤12 mm resistant; for nitrofurantoin, an inhibition zone diameter of ≥17 mm was considered susceptible, 15−16 mm intermediate and ≤14 mm resistant. *E. coli* ATCC 25922 (American Type Culture Collection, Manassas, VA, USA) was used as the control strain in the susceptibility assays.

Furthermore, in order to identify *E. coli* isolates producing fosfomycin resistance-mediating glutathione S-transferases, 20 μL sodium phosphonoformate (PPF) (Sigma-Aldrich, Madrid, Spain) was added at a concentration of 50 mg/mL on a second disk with 200 μg fosfomycin supplemented with 50 μg G6P, located at a distance of 30–35 mm from the first. After overnight incubation at 36 ± 1 °C, diameters of the growth inhibition zone were compared between the first disk (with PPF) and the second (without PPF). A difference of ≥5 mm between diameters was considered to confirm the phenotypic presence of the enzyme [8]. All assays were performed in duplicate.

### 2.2. Carbohydrate Utilization Test

All isolates were studied to determine the capacity for bacterial growth in the presence of a single source of carbon, *sn*-glycerol 3-phosphate (G3P), or G6P, using a previously described procedure [9]. After incubating bacteria in Mueller–Hinton broth for 24 h at 36 ± 1 °C in agitation, they were collected by centrifugation and resuspended in normal saline solution (0.9% NaCl). After five washes (to remove any remains that may act as carbon source), bacterial suspensions were then streaked onto M9 minimal medium agar supplemented with glucose (as a positive growth control), with G3P or G6P at 0.2% (w/v). Bacterial growth was determined after incubation at 36 ± 1 °C for 48 h. All assays were performed in duplicate. The absence of bacterial growth or poor growth with no colony formation in media supplemented with G3P or G6P was considered to indicate GlpT or UhpT function deficiency, respectively [9].

### 2.3. PCR Amplification

Polymerase chain reaction (PCR) was used to amplify *murA*, *glpT*, *uhpT*, *uhpA*, *ptsI*, *cyaA*, *nfsA*, *nfsB*, and *ribE* genes of *E. coli*, using previously reported procedures [5,9], separately amplifying two fragments (*cyaA1* and *cyaA2*) for the *cyaA* gene. The primer pairs used are listed in Table 1. DNA was obtained from clinical isolates and *E. coli* ATCC 25922 (used as control strain) using the PureLink Microbiome DNA Purification Kit (Thermo Fisher Scientific, Waltham, MA, USA). One microliter of the purified DNA was added to a master mix containing PCR buffer (1×), MgCl_2_ (2 mM), dNTPs (0.4 mM), primers (0.4 μM), and Taq polymerase (1.25 U).

PCR amplification of *murA*, *glpT*, *uhpT*, *cyaA1*, *cyaA2*, *nfsA*, *nfsB*, and *ribE* genes was performed as follows: 2 min of denaturation at 94 °C, followed by 35 cycles of denaturation at 94 °C for 30 s, annealing at 55 °C for 30 s and extension at 72 °C for 2 min (1 min for *nfsA*, *nfsB*, and *ribE*), with a final period of extension at 72 °C for 5 min. The same conditions were used for the amplification of *uhpA* and *ptsI* genes except that the annealing temperature was 57 °C.

For the isolates in which *uhpT* or *uhpA* genes could not be detected by the aforementioned procedure, a new PCR was designed using an outer primer pair (*uhpT*-F_2_: 5′-GATGTTAATCGGTATGGCGGC-3′; *uhpT*-R_2_: 5′-CAGTCGCTGGCGGAACAAAT-3′; *uhpA*-F_2_: 5′-CGTAATTCTGGAGCTCACCG-3′; *uhpA*-R_2_: 5′-CGCCTGCGTTAGCCAGTAA-3′). Besides re-amplification with outer primers, the amplification specificity was increased by using the forward outer primer with the reverse inner primer and the forward inner primer with the reverse outer primer.

Plasmid-borne fosfomycin resistance genes *fosA3*, *fosA4*, *fosA5*, *fosA6*, and *fosC2* and nitrofurantoin resistance genes *oqxA* and *oqxB* were screened by PCR amplification with the primers listed in Table 1, following previously reported procedures [8,10,11,12]. 

All PCR products were separated in 0.8% agarose gel and visualized under UV light after staining with ethidium bromide.

### 2.4. Nucleotide Sequencing 

Pools of 8 and 10 amplicons were established, and each amplicon was equimolarly normalized in the pool. Each pool was tagmented (tagged and fragmented) using the Nextera XT transposome, which fragments the DNA and then tags it with adapter sequences in a single step. The tagmented DNA was amplified with 12 PCR cycles. The PCR step also adds index 1 (i7), index 2 (i5), and full adapter sequences required for cluster formation. Each DNA sample was purified using 30 µL of AMPure XP beads and was resuspended in 50 μL of water. Then, it was quantified using a Qubit^®^ 3.0 Fluorometer (Thermo Fisher Scientific) and normalized. Pools were sequenced in a high cartridge of 300 cycles using a NextSeq platform. Data were mapped against the reference sequence of *E. coli* str. K-12 substr. MG1655 (NCBI Reference Sequence: NC_000913.3). A BAM file was generated, followed by a variant calling, and the most representative variants were recorded. The online Protein Variation Effect Analyzer (PROVEAN) platform (http://provean.jcvi.org/index.php) was used to predict the impact of identified amino acid substitutions on the biological function of each protein [13]. PROVEAN is able to provide predictions for any type of protein sequence variation, including single or multiple amino acid substitutions, insertions, or deletions. The platform introduces a delta alignment score based on the reference and variant versions of a protein query sequence with respect to sequence homologs collected from the NCBI protein database through BLAST. If the PROVEAN score (P-score) was equal to or below a predefined cutoff of −2.5, the protein variant was predicted to have a "deleterious" effect (potential loss of protein structure or function). If the P-score was above the threshold, the variant was predicted to have a "neutral" effect (no alteration in the structure or function of the protein).

## 3. Results

**Figure 1:**
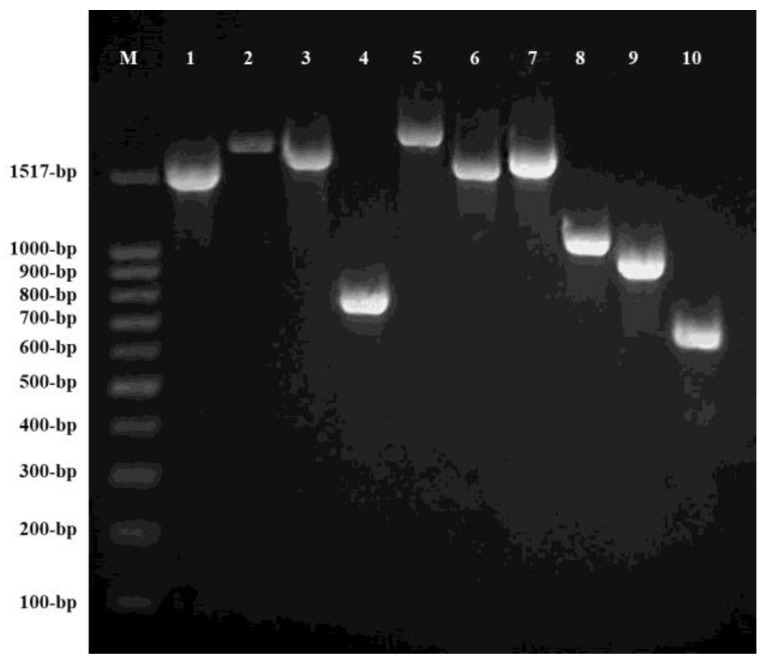
Electrophoresis results of polymerase chain reaction (PCR) products in *Escherichia coli* ATCC 25922 on 0.8% agarose gel. M: Molecular weight. Lines 1 to 10: PCR products of *murA* (1542 bp), *glpT* (1785 bp), *uhpT* (1667 bp), *uhpA* (771 bp), *ptsI* (1908 bp)*, cyaA1* (1559 bp), *cyaA2* (1648 bp), *nfsA* (1036 bp), *nfsB* (923 bp), and *ribE* (634 bp), respectively.

A total of 29 fosfomycin- and/or nitrofurantoin-resistant clinical isolates were identified: 8 were resistant to both fosfomycin and nitrofurantoin, 14 were resistant to fosfomycin and susceptible to nitrofurantoin, and 7 were susceptible to fosfomycin and resistant to nitrofurantoin. Figure 1 depicts PCR amplification of chromosomal genes *murA*, *glpT*, *uhpT*, *uhpA*, *ptsI*, *cyaA* (cyaA1 and cyaA2), *nfsA*, *nfsB*, and *ribE* in *E. coli* ATCC 25922.

### 3.1. Fosfomycin Resistance

Table 2 summarizes the characteristics of the 22 fosfomycin-resistant (inhibition zone diameter ≤12 mm around the disk with 200 μg fosfomycin supplemented with 50 μg glucose-6-phosphate) and 7 fosfomycin-susceptible (inhibition zone diameter ≥16 mm around the disk with 200 μg fosfomycin supplemented with 50 μg glucose-6-phosphate) clinical isolates of *E. coli* according to the CLSI procedure, displaying the diameter of the bacterial growth inhibition in the presence of PPF, the bacterial growth capacity in the presence of G3P or G6P as sole carbon source, and the amino acid substitutions in MurA, GlpT, UhpT, UhpA, PtsI, and CyaA proteins detected in each isolate. 

Three of the twenty-two fosfomycin-resistant isolates (strains 789, 809, and 853) showed a single substitution in the amino acid sequence of MurA (Leu370Ile), categorized as neutral (no alteration in structure or function of the protein) in the PROVEAN analysis (P-score: −1.995). 

Twelve amino acid substitutions were detected in GlpT: Glu448Lys (P-score: 0.486, categorized as neutral), in all isolates, both resistant and susceptible; Ala16Thr (P-score: −0.713, categorized as neutral), and Phe133Cys (P-score: −5.549), Gly135Trp (P-score: −7.756), Ala197Val (P-score: −3.472), and Leu373Arg (P-score: −5.328), all categorized as deleterious (potential loss of protein structure or function), in susceptible isolates alone; and Met52Leu (P-score: −1.261), Leu297Phe (P-score: −2.375), Glu443Gln (P-score: 0.014), and Gln444Glu (P-score: −0.106), all four categorized as neutral, and Gly84Asp (P-score: −6.056) and Pro212Leu (P-score: −9.698), both categorized as deleterious, in resistant isolates alone. 

No amplification product of the *uhpT* gene was obtained from strains 11 and 26 using the two primer pairs reported above (loss of entire gene). Amino acid substitution in UhpT (Glu350Gln), categorized as neutral (P-score: −0.016), was observed in 16 of the 22 fosfomycin-resistant isolates but in none of the susceptible isolates. 

The *uhpA* gene was detected in all fosfomycin-susceptible isolates, and two of these showed substitution of Arg46Cys in the protein sequence, categorized as neutral (P-score: −0.268). By contrast, this gene was detected in only 1 of the 22 fosfomycin-resistant isolates: strain 26 (wild-type). 

Three amino acid substitutions were detected in PtsI: Arg367Lys (P-score: 0.842), in all isolates, both resistant and susceptible; Ala306Thr (P-score: 0.030), in two susceptible isolates alone; and Val25Ile (P-score: −0.606), in 10 resistant isolates alone; and all three substitutions were categorized as neutral in the PROVEAN analysis. 

Finally, 11 amino acid substitutions were detected in CyaA: Asn142Ser (P-score: 0.016, categorized as neutral), in all isolates; Gly222Ser (P-score: −3.447, categorized as deleterious), in one of the seven susceptible isolates; Ala349Glu (P-score: 2.261), Glu362Asp (P-score: −0.286), Asp837Glu (P-score: 0.123), and Thr840Ala (P-score: −0.314) all four categorized as neutral; and Ser356Leu (P-score: −2.624) and Gly359Glu (P-score: −3.077) both categorized as deleterious, in some susceptible and resistant isolates; and Ala363Ser (P-score: 0.900), Ala363Gly (P-score: −0.251), and Ser352Thr (P-score: −0.645) all three categorized as neutral, in fosfomycin-resistant isolates alone.

The effect of these amino acid substitutions on transporters GlpT and UhpT in resistant and susceptible isolates was evaluated by testing bacterial growth on M9 minimal medium agar supplemented with G3P or G6P (substrates for GlpT or UhpT, respectively). As reported in Table 2, all isolates grew on M9 medium with G3P, indicating no significant loss of GlpT function with any substitution detected in the amino acid sequence of this transporter. However, fosfomycin-resistant isolates did not grow or showed poor growth on the medium containing G6P, because of the loss of function of UhpT due to the complete deletion of *uhpT* (strains 11 and 26) and/or *uhpA* genes (strains 11, 17, 66, 381, 387, 462, 632, 752, 757, 776, 789, 792, 795, 799, 809, 853, 854, 860, 871, 883 and 891). 

**Figure 2:**
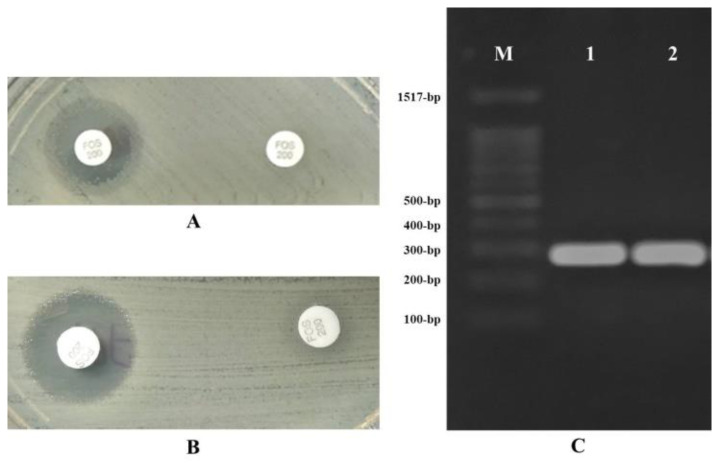
Detection of fosfomycin resistance-mediating glutathione S-transferase (sodium phosphonoformate test) and *fosA3* gene (electrophoresis) in strains 66 and 871. (**A**,**B**) Phenotypic detection of fosfomycin resistance-mediating glutathione S-transferase in strains 66 and 871, respectively, showing an increase of ≥5 mm in growth inhibition halo around the disk of 200 μg fosfomycin supplemented with 50 μg G6P plus sodium phosphonoformate in comparison to the disk containing 200 μg fosfomycin supplemented with 50 μg G6P alone. All assays were performed in duplicate in all isolates, obtaining the same between-assay results; (**C**) Electrophoresis results for the PCR products of *fosA3* gene (282 bp) on 0.8% agarose gel in strains 66 (line 1) and 871 (line 2). M: molecular weight.

Furthermore, fosfomycin resistance-mediating glutathione S-transferase was observed in two of the fosfomycin-resistant isolates (strains 66 and 871) due to a significantly increased bacterial growth inhibition halo (≥5 mm) in the presence of PPF (Table 2). This phenotypic finding was confirmed by PCR amplification of the *fosA3* gene in both isolates (Figure 2). No *fosA4*, *fosA5*, *fosA6*, and *fosC2* plasmid genes were detected in any isolate.

### 3.2. Nitrofurantoin Resistance

Table 3 summarizes the characteristics of the 15 nitrofurantoin-resistant or intermediate isolates (inhibition zone diameter ≤14 mm or 13−15 mm, respectively, around the disk with 300 μg nitrofurantoin) and the 14 nitrofurantoin-susceptible (inhibition zone diameter ≥17 mm around the disk with 300 μg nitrofurantoin) clinical isolates of *E. coli* according to the CLSI procedure. Amino acid substitutions in NfsA, NfsB, or RibE proteins were detected in all isolates. 

Among the 15 nitrofurantoin-resistant or nitrofurantoin-intermediate and 14 nitrofurantoin-susceptible isolates, 14 amino acid substitutions were detected in the NfsA protein: Glu58Asp (P-score: −1.866), Ile117Thr (P-score: −0.634), Lys141Glu (P-score: 1.207), Gln147Arg (P-score: −1.170), and Gly187Asp (P-score: 1.554) all of these categorized as neutral in the PROVEAN analysis (no alteration in structure or function of the protein), in susceptible isolates; Asp19Asn (P-score: −2.091) and Ser180Asn (P-score: 0.071) both categorized as neutral, and His11Tyr (P-score: −5.746), Ser33Arg (P-score: −2.526), Gln67Leu (P-score: −5.860), Cys80Arg (P-score: −11.148), Gly126Arg (P-score: −7.544), Gly154Glu (P-score: −7.608), and Arg203Cys (P-score: −7.090) all of these categorized as deleterious (potential loss of protein structure or function), only in isolates with some level of resistance (resistant or intermediate). In addition, a single nucleotide mutation in the *nfsA* gene was detected in strains 757 and 802, leading to truncation of the NfsA sequence in Gln67 (Gln67stop; CAA to TAA) and Gln147 (Gln147stop; CAG to TAG), respectively. These mutations produced 66 and 146 amino acid long proteins, respectively, instead of a wild-type protein with 240 amino acids.

Eleven amino acid substitutions were detected in NfsB: Gly66Asp (P-score: −1.775), Val93Ala (P-score: 2.155), and Ala174Glu (P-score: 1.621) all of these categorized as neutral, in susceptible isolates; and Leu22Ile (P-score: 0.334), Met75Ile (P-score: 2.094), and Lys122Arg (P-score: −0.179) all of these categorized as neutral, and Gln44His (P-score: −4.800), Phe84Ser (P-score: −5.862), Arg107Cys (P-score: −7.863), Gly192Ser (P-score: −5.961), and Arg207His (P-score: −4.966) all of these categorized as deleterious, in isolates with some level of resistance (resistant or intermediate). In addition, a single nucleotide mutation in the *nfsB* gene (GAA to TAA) was detected in strain 11, leading to a truncation of the NfsB sequence in Glu54.

Two amino acid substitutions in RibE were detected in three isolates with some level of resistance: Val51Ile (P-score: −0.363, categorized as neutral) and Pro55His (P-score: −8.840, categorized as deleterious). Finally, no *oqxAB* plasmid gene was detected in any isolate.

## 4. Discussion

### 4.1. Mechanisms of Resistance to Fosfomycin in E. coli

Mechanisms of resistance to fosfomycin described in various bacteria include the modification or overexpression of target molecule MurA, a reduced permeability, and irreversible antibiotic modification. The first two mechanisms are chromosomal, whereas the third can be chromosomal or encoded in transferable multi-resistance plasmids [14].

#### 4.1.1. Modification or Overexpression of the Target (MurA)

The main action mechanism of fosfomycin is inhibition of the first step of peptidoglycan synthesis. Its chemical structure is analogous to that of phosphoenolpyruvate (PEP), therefore blocking the active center of enzyme UDP-N-acetylglucosamine enolpyruvyl transferase (MurA), covalently binding to the residue of cysteine Cys115 and preventing the binding of the substrate with the enzyme. In *E. coli*, amino acid substitutions in the active center of MurA, specifically Cys115Asp, are related to fosfomycin resistance [15] but are not common in clinical isolates of this species due to a drastic reduction in bacterial cell viability [16]. Only a few reports have associated amino acid substitutions in the MurA sequence of *E. coli* with resistance, especially Asp369Asn and Leu370Ile [9]. The latter was detected in 3 of the 22 fosfomycin-resistant isolates in the present study (strains 789, 809, and 853) but the protein variant was predicted to have a neutral effect in the PROVEAN analysis, with no alteration in the structure or function of the protein. Although previous crystallization studies found that leucine in position 370 of MurA does not interfere with its binding to fosfomycin, the fact that it is a highly preserved residue suggests an important role in the binding of PEP and therefore fosfomycin to the active site of the enzyme [9].

#### 4.1.2. Permeability Reduction 

Fosfomycin can use two transport systems to access the bacterial cytoplasm: glycerol-3-phosphate transporter (GlpT) and hexose phosphate transporter (UhpT). They are induced by the presence of their substrates (G3P and G6P, respectively) and require high levels of cyclic AMP (cAMP), whose synthesis depends on the enzyme adenylate cyclase (CyaA) and is regulated by the phosphoenolpyruvate-protein phosphotransferase (PtsI) system. The expression of GlpT is determined by a repressor gene, *glpR*, given that the interaction of GlpR with G3P increases transcription of the *glpT* gene. The expression of UhpT is in turn controlled by various regulating genes (*uhpA*, *uhpB*, and *uhpC*) [14]. This mechanism of action is unique; it does not confer cross-resistance to other antibiotics and it favors additive action with beta-lactams, aminoglycosides, glycopeptides, and fluoroquinolones, among others [17].

GlpT and UhpT are transporters with an extensive amino acid sequence homology that appear in several bacterial species with a high degree of conservation [14]. Various studies of *E. coli* have identified modifications of these proteins and/or proteins that regulate their expression (UhpA, PtsI, and CyaA) due to gene mutations or complete loss [9,18,19,20,21]. However, although the most important fosfomycin-resistance mechanism in this bacterium, modifications in chromosomal genes *uhpT*, *glpT*, *uhpA*, *ptsI*, or *cyaA* are reported to carry a high fitness cost, and clinical isolates with this resistance are known to be outcompeted by isolates susceptible to fosfomycin [22].

In the present study, all clinical isolates of *E. coli* presented substitutions in the amino acid sequence of GlpT. Some of them were detected in fosfomycin-susceptible isolates (Ala16Thr, Phe133Cys, Gly135Trp, Ala197Val, Leu373Arg, and Glu448Lys). Hence, these substitutions do not appear to be related per se to an alteration in GlpT function or resistance to the antibiotic. Other substitutions were solely detected in resistant isolates (Met52Leu, Leu297Phe, Glu443Gln, and Gln444Glu) but were classified as neutral in the PROVEAN analysis and would have no impact on the biological function of this protein. According to the PROVEAN analysis, only Gly84Asp and Pro212Leu substitutions could be significantly related to an alteration of GlpT functionality; however, the two isolates with this substitution (strains 26 and 752) proved able to grow in the presence of G3P. Hence, all isolates grew on M9 medium with G3P, indicating no significant loss of GlpT function with any substitution detected in the amino acid sequence of this transporter.

Among the 22 fosfomycin-resistant *E. coli* isolates, 4 showed no amino acid substitution in UhpT, 16 showed one substitution (Glu350Gln) and 2 were defective in UhpT due to gene loss (strains 11 and 26). We highlight that the *uhpA* gene was detected in strain 26 alone and that none of the 22 fosfomycin-resistant isolates were able to grow in the presence of G6P. In the UhpA sequence, the only substitution was Arg46Cys, which was only detected in two fosfomycin-susceptible isolates; therefore, it does not appear to be related per se to an alteration in the function of these proteins or to antibiotic resistance. According to our findings, all of the resistant isolates analyzed were defective in the UhpT transport system due to *uhpT* and/or *uhpA* deletion and showed no growth or only poor growth in a medium containing G6P as sole carbon source. Therefore, this finding supports the hypothesis that fosfomycin resistance in *E. coli* is most frequently attributable to blockage of the entry pathway of the antibiotic into the bacteria, mainly due to modifications in the UhpT transporter or its regulating proteins [18,19]. 

All of the *E. coli* clinical isolates in the present study showed substitutions in PtsI and CyaA. Given that some of these were detected in fosfomycin susceptible isolates (Ala306Thr and Arg367Lys in PtsI; Asn142Ser, Gly222Ser, Ala349Glu, Ser356Leu, Gly359Glu, Glu362Asp, Asp837Glu, and Thr840Ala in CyaA), they do not appear to be related per se to an alteration in the function of these proteins or to antibiotic resistance. Some other substitutions in these proteins were only detected in resistant isolates (Val25Ile in PtsI; Ser352Thr, Ala363Ser, and Ala363Gly in CyaA), as in previous studies [9]; nevertheless, their contribution to antibiotic resistance in these isolates cannot be affirmed, given that they were categorized as neutral in the PROVEAN analysis and there was no alteration in the function of GlpT, which was permeable to G3P. Therefore, it cannot be affirmed that amino acid substitutions in PtsI and CyaA contributed to resistance to fosfomycin in the clinical isolates of *E. coli* in the present study.

#### 4.1.3. Enzymatic Modification of Fosfomycin

Two mechanisms may underlie fosfomycin resistance due to the action of modifying enzymes: epoxide ring opening, catalyzed by FosA enzymes (glutathione S-transferase), FosB (L-cysteine thiol transferase), or FosX (hydrolase epoxide); or antibiotic phosphorylation by FomA, FomB, or FosC enzymes [23]. Among these enzymes, FosA3 is the most widely described in *E. coli* plasmids, largely in Eastern Asia countries, although its detection is infrequent in Europe [11]. To our knowledge, this is the first time that the *fosA3* gene has been detected in clinical isolates of *E. coli* in Spain (strains 66 and 871). Both isolates were also defective in the UhpT transport system due to *uhpA* deletion; however, the importance of this finding is that this plasmid-mediated gene may accelerate the dissemination of fosfomycin resistance in the near future.

### 4.2. Mechanisms of Resistance to Nitrofurantoin in E. coli

Nitrofurantoin is a prodrug of the nitrofuran family and exerts its antibiotic activity via multiple mechanisms of action, although none have been fully elucidated. It is known to inhibit: (i) protein synthesis, (ii) aerobic metabolism, (iii) nucleic acid synthesis, and (iv) cell wall synthesis. Its active form is generated within the bacterium by the action of nitroreductase enzymes, which reduce the nitro group coupled to the furan heterocyclic ring, giving rise to active intermediate metabolites that inhibit the synthesis of proteins involved in DNA, RNA, and carbohydrate metabolism. 

Various studies have attributed resistance to nitrofurantoin in *E. coli* to the loss of intracellular nitroreductase activity via sequential mutations in *nfsA* and *nfsB* genes, which encode oxygen-insensitive nitroreductases, as well as to deletions affecting the active center of *ribE*, although the latter have not yet been reported in clinical isolates. Mutations in genes encoding oxygen-sensitive nitroreductases have not yet been described [5,24]. However, as in the case of fosfomycin, this nitrofurantoin resistance is reported to confer a high biological cost, and clinical isolates with this resistance are known to be outcompeted by susceptible isolates, reducing the likelihood of its detection in clinical isolates [5].

All *E. coli* clinical isolates in the present study showed substitutions in the amino acid sequence of NfsA and/or NfsB. As reported above, some were detected in nitrofurantoin-susceptible isolates (Glu58Asp, Ile117Thr, Lys141Glu, Gln147Arg, and Gly187Asp in NfsA; and Gly66Asp, Val93Ala, and Ala74Glu in NfsB). Although some of these (positions Ile117 and Lys141 in NfsA; Gly66 and Val93 in NfsB) have been associated with resistance in other studies [5,24,25], they were all classified as neutral in the PROVEAN analysis. Hence, none of these substitutions appear to be related per se to an alteration in the function of these proteins or to resistance to the antibiotic.

Other substitutions were detected in resistant isolates alone (His11Tyr, Asp19Asn, Ser33Arg, Gln67Leu, Cys80Arg, Gly126Arg, Gly154Glu, Ser180Asn, Arg203Cys, and truncation at Gln67 and Gln147 in NfsA; Leu22Ile, Gln44His, Met75Ile, Phe84Ser, Arg107Cys, Lys122Arg, Gly192Ser, Arg207His, and truncation at Glu54 in NfsB; Val51Ile and Pro55His in RibE). Some of these (His11, Ser33, Gln67, Gln147, and Arg203 in NfsA; Gln44, Met75, Arg107, Lys122, Gly192, and Arg207 in NfsB) have been associated with nitrofurantoin resistance in other studies [5,24,25,26]. According to the PROVEAN analysis, His11Tyr, Ser33Arg, Gln67Leu, Cys80Arg, Gly126Arg, Gly154Glu, and Arg203Cys in NfsA; Gln44His, Phe84Ser, Arg107Cys, Gly192Ser, and Arg207His in NfsB; and Pro55His in RibE were predicted to have a deleterious impact on the protein structure. Production of truncated NfsA (Gln67 and Gln147) or NfsB (Glu54) may have resulted in the inability or reduced ability of nitrofurantoin-resistant isolates to reduce the nitrofurantoin and produce active intermediates from the compound. Hence, these amino acid substitutions and/or truncated proteins would be related to nitrofurantoin resistance.

According to various studies, NfsA inactivation followed by NfsB inactivation is the main mechanism for high-level nitrofurantoin resistance in *E. coli* [5,26]. However, several of our nitrofurantoin-resistant clinical isolates did not show any modification in the NfsA sequence compatible with resistance (strains 66, 302, 799, 854, 883, and 892). Among these six isolates, we only detected substitutions in the NfsB sequence compatible with resistance (Arg207His) in the first two. However, we cannot affirm its association with resistance in the other four, although they presented various amino acid substitutions. Therefore, the mechanism that produces nitrofurantoin resistance in these four isolates is yet to be elucidated. Some authors have affirmed that NfsB inactivation in the presence of a wild-type *nfsA* gene cannot be associated with resistance [26]; in contrast, according to our findings, certain NfsB modifications requiring no previous NfsA alterations may be responsible for the functional alteration of bacterial nitroreductases, as also previously reported [24]. 

More recently, it has been reported that the presence of OqxAB (a plasmid-encoded multidrug efflux pump that confers reduced susceptibility to quinolones, tigecycline, chloramphenicol, trimethoprim, and disinfectants such as quaternary ammonium compounds) would also enhance nitrofurantoin resistance via an active antibiotic expulsion mechanism in *E. coli* isolates with previous nitroreductase modifications, because it has not been possible to relate the presence of OqxAB per se in the bacterium to antibiotic resistance levels [27]. This plasmid has been widely detected in *E. coli* and other enterobacteria, both in human and animal isolates, mainly in China [25,27]; although its presence has also been reported in Europe [28,29], including Spain [30]. However, this plasmid was not detected in any of our series of isolates, indicating that nitrofurantoin resistance must involve mechanisms other than antibiotic extrusion.

Finally, as in the present study, there have been reports of nitrofurantoin-resistant *E. coli* isolates with no amino acid substitutions in NfsA, NfsB, and RibE, or presence of the *oqxAB* plasmid, indicating the need to identify new mechanisms that explain nitrofurantoin resistance in this bacterium [27]. 

## 5. Conclusions

These results suggest that the emergence of fosfomycin resistance in clinical isolates of *E. coli* in our setting is largely attributable to the absence of expression of transporter UhpT due to complete deletion of the *uhpT* and/or *uhpA* regulating genes, reducing the permeability of the bacterium to the antibiotic. To our knowledge, we report for the first time the presence in Spain of the plasmid gene *fosA3*, responsible for the enzyme glutathione S-transferase, which inactivates the antibiotic. We consider this finding to be of major epidemiological importance, given its potential dissemination not only in *E. coli* but also other bacteria. Nitrofurantoin resistance can be explained, at least in part, by the presence of specific modifications in NfsA, NfsB, or RibE proteins. The presence of *oqxAB* plasmid genes does not appear to represent an important resistance mechanism among *E. coli* clinical isolates in our setting at the present time. The emergence and spread of these resistance mechanisms, including transferable resistance, could compromise the future usefulness of fosfomycin and nitrofurantoin against UTIs. 

## Figures and Tables

**Table 1:** Primers used for amplification and sequencing of the *Escherichia coli* genes involved in fosfomycin or nitrofurantoin resistance.

Gene	Forward Primer	Reverse Primer	Amplicon Size (bp)	Reference
*murA*	5′-AAACAGCAGACGGTCTATGG-3′	5′-CCATGAGTTTATCGACAGAACG-3′	1542	[9]
*glpT*	5′-GCGAGTCGCGAGTTTTCATTG-3′	5′-GGCAAATATCCACTGGCACC-3′	1785	
*uhpT*	5′-TTTTTGAACGCCCAGACACC-3′	5′-AGTCAGGGGCTATTTGATGG-3′	1667	
*uhpA*	5′-GATCGCGGTGTTTTTTCAG-3′	5′-GATACTCCACAGGCAAAACC-3′	771	
*ptsI*	5′-GAAAGCGGTTGAACATCTGG-3′	5′-TCCTTCTTGTCGTCGGAAAC-3′	1908	
*cyaA1*	5′-AACCAGGCGCGAAAAGTGG-3′	5′-TGATGGCTGATGATCGACTC-3′	1559	[9] This study
*cyaA2*	5′-AAAGCTCAGCCGTGAACGC-3′	5′-ACCTTCTGGGATTTGCTGG-3′	1648	
*nfsA*	5′-ATTTTCTCGGCCAGAAGTGC-3′	5′-AGAATTTCAACCAGGTGACC-3′	1036	[5]
*nfsB*	5′-CTTCGCGATCTGATCAACG-3′	5′-CAACAGCAGCCTATGATGAC-3′	923	
*ribE*	5′-AAGGGAAGCAGCGCACGAA-3′	5′-GGACAACTGCCAGGAGTAGA-3′	634	This study
*fosA3*	5′-GCGTCAAGCCTGGCATTT-3′	5′-GCCGTCAGGGTCGAGAAA-3′	282	[10]
*fosA4*	5′-CTGGCGTTTTATCAGCGGTT-3′	5′-CTTCGCTGCGGTTGTCTTT-3′	230	[11]
*fosA5*	5′-TATTAGCGAAGCCGATTTTGCT-3′	5′-CCCCTTATACGGCTGCTCG-3′	177	
*fosA6*	5′-GCTACGGTTCAGCTTCCAGA-3′	5′-CGAGCGTGGCGTTTTATCAG-3′	242	This study
*fosC2*	5′-CGTTCCGTGGAGTTCTATAC-3′	5′-CTTGATAGGGTTTAGACTTC-3′	334	[8]
*oqxA*	5′-GACAGCGTCGCACAGAATG-3′	5′-GGAGACGAGGTTGGTATGGA-3′	339	[12]
*oqxB*	5′-CGAAGAAAGACCTCCCTACCC-3′	5′-CGCCGCCAATGAGATACA-3′	240	

**Table 2:** Susceptibility to fosfomycin according to the Clinical and Laboratory Standards Institute (CLSI) procedure and supplemented with sodium phosphonoformate (PPF); bacterial growth on M9 minimal medium agar supplemented with *sn*-glycerol 3-phosphate (G3P) or glucose-6-phosphate (G6P); and amino acid substitutions in MurA, GlpT, UhpT, UhpA, PtsI, and CyaA proteins in 29 clinical isolates of *Escherichia coli*.

Strain	Fosfomycin Disk ^1^	Clinical Category ^2^	Fosfomycin Disk Plus PPF ^3^	G3P ^4^	G6P ^5^	Amino Acid Substitutions in					
						MurA	GlpT	UhpT	UhpA	PtsI	CyaA
11	6	R	6	+	−	None	Leu297Phe Glu443Gln Gln444Glu Glu448Lys	Not detected	Not detected	Arg367Lys	Asn142Ser Ala349Glu Ser352Thr Ser356Leu Gly359Glu Glu362Asp
17	6	R	6	+	−	None	Glu448Lys	Glu350Gln	Not detected	Val25Ile Arg367Lys	Asn142Ser Asp837Glu Thr840Ala
26	12	R	12	+	− ^a^	None	Gly84Asp Glu448Lys	Not detected	None	Arg367Lys	Asn142Ser Ala349Glu Ser356Leu Gly359Glu Glu362Asp Ala363Ser Asp837Glu Thr840Ala
66	6	R	13	+	−	None	Glu448Lys	Glu350Gln	Not detected	Val25Ile Arg367Lys	Asn142Ser Asp837Glu Thr840Ala
302	29	S	30	+	+	None	Glu448Lys	None	None	Arg367Lys	Asn142Ser Gly222Ser
334	31	S	32	+	+	None	Glu448Lys	None	None	Arg367Lys	Asn142Ser
381	6	R	6	+	−	None	Glu448Lys	Glu350Gln	Not detected	Val25Ile Arg367Lys	Asn142Ser Asp837Glu Thr840Ala
387	6	R	6	+	−	None	Glu448Lys	Glu350Gln	Not detected	Val25Ile Arg367Lys	Asn142Ser Asp837Glu Thr840Ala
462	12	R	13	+	−	None	Glu448Lys	Glu350Gln	Not detected	Val25Ile Arg367Lys	Asn142Ser Asp837Glu Thr840Ala
632	6	R	6	+	−	None	Leu297Phe Glu443Gln Gln444Glu Glu448Lys	Glu350Gln	Not detected	Arg367Lys	Asn142Ser Ala349Glu Ser352Thr Ser356Leu Gly359Glu Glu362Asp Ala363Gly
751	30	S	32	+	+	None	Ala16Thr Glu448Lys	None	Arg46Cys	Ala306Thr Arg367Lys	Asn142Ser Ala349Glu Ser356Leu Gly359Glu Glu362Asp Asp837Glu Thr840Ala
752	11	R	11	+	−	None	Pro212Leu Glu448Lys	Glu350Gln	Not detected	Val25Ile Arg367Lys	Asn142Ser Asp837Glu Thr840Ala
757	6	R	6	+	− ^a^	None	Leu297Phe Glu443Gln Gln444Glu Glu448Lys	None	Not detected	Arg367Lys	Asn142Ser Ala349Glu Ser352Thr Ser356Leu Gly359Glu Glu362Asp
776	12	R	12	+	− ^a^	None	Glu448Lys	Glu350Gln	Not detected	Val25Ile Arg367Lys	Asn142Ser Asp837Glu Thr840Ala
789	6	R	6	+	−	Leu370Ile	Leu297Phe Glu443Gln Gln444Glu Glu448Lys	Glu350Gln	Not detected	Arg367Lys	Asn142Ser Ala349Glu Ser352Thr Ser356Leu Gly359Glu Glu362Asp
792	6	R	6	+	−	None	Glu448Lys	Glu350Gln	Not detected	Val25Ile Arg367Lys	Asn142Ser Asp837Glu Thr840Ala
795	6	R	6	+	−	None	Leu297Phe Glu443Gln Gln444Glu Glu448Lys	Glu350Gln	Not detected	Arg367Lys	Asn142Ser Ala349Glu Ser352Thr Ser356Leu Gly359Glu Glu362Asp
797	20	S	21	+	+	None	Ala16Thr Leu373Arg Glu448Lys	None	Arg46Cys	Ala306Thr Arg367Lys	Asn142Ser Ala349Glu Ser356Leu Gly359Glu Glu362Asp Asp837Glu Thr840Ala
799	6	R	7	+	−	None	Leu297Phe Glu443Gln Gln444Glu Glu448Lys	None	Not detected	Arg367Lys	Asn142Ser Ala349Glu Ser352Thr Ser356Leu Gly359Glu Glu362Asp
802	30	S	30	+	+	None	Phe133Cys Gly135Trp Ala197Val Glu448Lys	None	None	Arg367Lys	Asn142Ser
809	6	R	6	+	−	Leu370Ile	Leu297Phe Glu443Gln Gln444Glu Glu448Lys	Glu350Gln	Not detected	Arg367Lys	Asn142Ser Ala349Glu Ser352Thr Ser356Leu Gly359Glu Glu362Asp
853	6	R	8	+	−	Leu370Ile	Leu297Phe Glu443Gln Gln444Glu Glu448Lys	Glu350Gln	Not detected	Arg367Lys	Asn142Ser Ala349Glu Ser352Thr Ser356Leu Gly359Glu Glu362Asp
854	6	R	6	+	−	None	Glu448Lys	None	Not detected	Arg367Lys	Asn142Ser
860	6	R	7	+	−	None	Met52Leu Leu297Phe Glu443Gln Gln444Glu Glu448Lys	None	Not detected	Arg367Lys	Asn142Ser Ala349Glu Ser352Thr Ser356Leu Gly359Glu Glu362Asp
871	6	R	14	+	− ^a^	None	Leu297Phe Glu443Gln Gln444Glu Glu448Lys	Glu350Gln	Not detected	Arg367Lys	Asn142Ser Ala349Glu Ser352Thr Ser356Leu Gly359Glu Glu362Asp Ala363Gly
872	35	S	35	+	+	None	Glu448Lys	None	None	Arg367Lys	Asn142Ser Asp837Glu Thr840Ala
883	11	R	12	+	−	None	Glu448Lys	Glu350Gln	Not detected	Val25Ile Arg367Lys	Asn142Ser Asp837Glu Thr840Ala
891	11	R	11	+	−	None	Glu448Lys	Glu350Gln	Not detected	Val25Ile Arg367Lys	Asn142Ser Asp837Glu Thr840Ala
892	21	S	21	+	+	None	Glu448Lys	None	None	Arg367Lys	Asn142Ser

^1^ Diameter (in mm) of the bacterial growth inhibition halo around the disk with 200 μg fosfomycin supplemented with 50 μg glucose-6-phosphate on Mueller-Hinton agar. ^2^ Clinical categories of each isolate against fosfomycin according to CLSI breakpoints (S: susceptible; R: resistant). ^3^ Diameter (in mm) of the bacterial growth inhibition halo around the disk with 200 μg fosfomycin supplemented with 50 μg glucose-6-phosphate and 20 μL sodium phosphonoformate (PPF) in order to identify *E. coli* isolates producing fosfomycin resistance-mediating glutathione S-transferases (between-diameter difference of ≥5 mm considered to confirm the phenotypic presence of the enzyme). ^4^ Bacterial growth on M9 minimal medium agar supplemented with 0.2% *sn*-glycerol 3-phosphate (all isolates showed growth). ^5^ Bacterial growth on M9 minimal medium agar supplemented with 0.2% glucose-6-phosphate (+: bacterial growth; −: absence of bacterial growth). ^a^ Only poor growth was observed after 48 h of incubation. Not detected: gene not detected by PCR after the different combinations of two pairs of primers (loss of the entire gene). None: no amino acid substitutions found.

**Table 3:** Susceptibility to nitrofurantoin according to the Clinical and Laboratory Standards Institute (CLSI) procedure and amino acid substitutions in NfsA, NfsB, or RibE proteins of 29 clinical isolates of *Escherichia coli*.

Strain	Nitrofurantoin Disk ^1^	Clinical Category ^2^	Amino Acid Substitutions in		
			NfsA	NfsB	RibE
11	11	R	Ile117Thr Gly126Arg Lys141Glu Gln147Arg Gly187Asp	Truncated at Glu54	None
17	27	S	Ile117Thr Lys141Glu Gly187Asp	Gly66Asp Val93Ala Ala174Glu	None
26	24	S	Ile117Thr Lys141Glu Gly187Asp	Gly66Asp Val93Ala Ala174Glu	None
66	14	R	Ile117Thr Lys141Glu Gly187Asp	Gly66Asp Val93Ala Ala174Glu Arg207His	None
302	14	R	None	Gly66Asp Met75Ile Val93Ala Ala174Glu Arg207His	None
334	15	I	Gln67Leu	Gly66Asp Met75Ile Val93Ala Arg107Cys	Pro55His
381	25	S	Ile117Thr Lys141Glu Gly187Asp	Gly66Asp Val93Ala Ala174Glu	None
387	21	S	Ile117Thr Lys141Glu Gly187Asp	Gly66Asp Val93Ala Ala174Glu	None
462	14	R	Cys80Arg Ile117Thr Lys141Glu Gly187Asp	Gly66Asp Val93Ala Ala174Glu Gly192Ser	None
632	20	S	Glu58Asp Ile117Thr Lys141Glu Gln147Arg Gly187Asp	Val93Ala	None
751	14	R	Ile117Thr Lys141Glu Gly187Asp Arg203Cys	Gly66Asp Val93Ala Ala174Glu	Val51Ile
752	22	S	Ile117Thr Lys141Glu Gly187Asp	Gly66Asp Val93Ala Ala174Glu	None
757	16	I	Glu58Asp Truncated at Gln67	Val93Ala Lys122Arg	None
776	19	S	Ile117Thr Lys141Glu Gly187Asp	Gly66Asp Val93Ala Ala174Glu	None
789	24	S	Glu58Asp Ile117Thr Lys141Glu Gln147Arg Gly187Asp	Val93Ala	None
792	25	S	Ile117Thr Lys141Glu Gly187Asp	Gly66Asp Val93Ala Ala174Glu	None
795	16	I	His11Tyr Glu58Asp Ile117Thr Lys141Glu Gln147Arg Gly187Asp	Val93Ala	None
797	12	R	Ile117Thr Lys141Glu Gly154Glu Gly187Asp	Leu22Ile Gly66Asp Val93Ala Ala174Glu	Val51Ile
799	13	R	Ile117Thr Lys141Glu Gln147Arg Gly187Asp	Val93Ala	None
802	12	R	Truncated at Gln147	Met75Ile Val93Ala	None
809	28	S	Glu58Asp Ile117Thr Lys141Glu Gln147Arg Gly187Asp	Val93Ala	None
853	20	S	Glu58Asp Ile117Thr Lys141Glu Gln147Arg Gly187Asp	Val93Ala	None
854	16	I	None	Gly66Asp Met75Ile Val93Ala	None
860	20	S	Ile117Thr Lys141Glu Gln147Arg Gly187Asp	Val93Ala	None
871	24	S	Glu58Asp Ile117Thr Lys141Glu Gln147Arg Gly187Asp	Val93Ala	None
872	15	I	Asp19Asn Ser33Arg Ile117Thr Lys141Glu Gly187Asp	Gln44His Gly66Asp Val93Ala Ala174Glu	None
883	13	R	Ile117Thr Lys141Glu Gly187Asp	Gly66Asp Phe84Ser Val93Ala Ala174Glu	None
891	23	S	Ile117Thr Lys141Glu Gly187Asp	Gly66Asp Val93Ala Ala174Glu	None
892	10	R	None	Gly66Asp Met75Ile Val93Ala	None

^1^ Diameter (in mm) of bacterial growth inhibition halo around the disk with 300 μg nitrofurantoin. ^2^ Clinical categories of each isolate against nitrofurantoin according to CLSI breakpoints (S: susceptible; I: intermediate; R: resistant). None: no amino acid substitutions were found.
